# Influence of the Surface Roughness of PEEK GRF30 and Ti6Al4V SLM on the Viability of Primary Human Osteoblasts Determined by the MTT Test

**DOI:** 10.3390/ma12244189

**Published:** 2019-12-13

**Authors:** Piotr Prochor, Żaneta Anna Mierzejewska

**Affiliations:** Institute of Biomedical Engineering, Faculty of Mechanical Engineering, Bialystok University of Technology, 15-351 Bialystok, Poland; a.mierzejewska@pb.edu.pl

**Keywords:** biomedical material, cytotoxicity, osteoblasts, sinister laser melting, surface roughness

## Abstract

The aim of the study was to clearly determine whether selected modern medical materials and three dimensional printing allow for satisfactory viability of human osteoblasts, which is important from the point of view of the subsequent osseointegration process. Moreover, as implants are produced with various topography, the influence of surface roughness on viability of bone cells was evaluated. To conduct the research, primary human osteoblasts (PromoCell) were used. Cells were seeded on samples of glass-reinforced polyetheretherketone (30% of the filling), Ti6Al4V manufactured with the use of selective laser melting technology and forged Ti6Al4V with appropriately prepared variable surface roughness. To assess the viability of the tested cells the 3-(4,5-dimethylthiazol-2-yl)-2,5-diphenyltetrazolium bromide was used. Results showed that all evaluated materials do not exhibit cytotoxic properties. Moreover, on their basis it can be concluded that there is a certain surface topography (designated i.a. as roughness equal to approx. Ra = 0.30 μm), which ensures the highest possible viability of human osteoblasts. On the basis of the received data, it can also be concluded that modern glass-reinforced polyetheretherketone or Ti6Al4V produced by rapid prototyping method allow to manufacture implants that should be effectively used in clinical conditions.

## 1. Introduction

One of the crucial tests evaluating the biocompatibility of medical materials is the cytotoxicity test, originally used by Mosmann, which assesses cells’ viability by reducing the 3-(4,5-dimethylthiazol-2-yl)-2,5-diphenyltetrazolium bromide (MTT) to an insoluble purple formazan [1,2]. The feature to convert tetrazolium salts to formazan is characteristic for metabolically active cells, as this process occurs due to oxidoreductive enzymes (primarily succinate dehydrogenase) in living mitochondria [3]. In the case of damaged or dead cells, this process proceeds with less intensity or does not take place completely [4]. For this reason, the quantity of product in the form of formazan crystals is proportional to the number of living cells that are being tested at a given moment [3]. After dissolution in appropriate solvents, the crystals have a maximum absorbance at wavelength (λ) of about 492–570 nm [5,6]. By spectrophotometric absorbance measurements of cell and its background, it is possible to determine cells’ viability [7]. The MTT test is widely used to assess the activity of epidermal or osteoblast cells [1,8].

Osteoblasts are a product of mesenchymal stem cells differentiation and are responsible for bone formation and its remodeling, while in in-vivo conditions they form, rich in ossein, type I collagen [9]. It is an organic intercellular substance, secreted by osteoblasts, that provides flexibility and endurance to a bone. During the mineralization process, ossein is transformed into bone plates, while during maturation osteoblasts undergo osteogenesis and form osteocytes [10]. Currently, Saos-2 cell line is systematically used to assess the biocompatibility of the examined materials with bone tissue [11]. These cells were firstly derived from an 11-year-old Caucasian girl suffering on osteosarcoma by Fogh in 1973 [12]. Saos-2 are characterized by features typical to osteoblasts, including similar process of differentiation, which allows their use as osteoblast-like cells to assess biocompatibility. Despite other substantial advantages, such as their common availability (low costs) or well- described properties, Saos-2 features some disadvantages, among which is the formation of the extracellular matrix that differs from the one created by normal osteoblasts, which may negatively influence the results of the carried out tests [13]. Currently, there are few studies using the primary human osteoblasts that would present actual biofunctionality of tested materials [14,15,16]. This is mainly due to their high costs as well as the long and relatively difficult process of their isolation [17].

Most of the currently performed MTT tests are carried out on porous surfaces applied to the previously prepared surface of the material [18,19]. Despite the positive effect of the porous layers, polished surfaces are still used (in the case of implants for direct skeletal attachment of limb prosthesis these surfaces find their use in some variants of the medullary part of the intraosseous transcutaneous amputation prosthesis system), which also allows us to obtain appropriate osseointegration [20].

Currently, there is a growing tendency to use orthopedic implants made of plastic composites, characterized by relatively small and similar stiffness to bone’s [21]. For this reason, polyetheretherketone (PEEK) composites are widely used due to their satisfying mechanical and biological features [22].

It is generally accepted, and confirmed by several experiments that PEEK is non-toxic material in in-vivo conditions [23,24,25]. However, current research on the biological properties of PEEK and its composites, with the use of MTT test, suggests that during its wear, friction products (small particles of broken and separated material) with a size of 0.23–2.00 μm may be formed, that can be characterized by certain toxicity to tissues, however which is acceptable in terms of its intensity. This suggests that the damaged surface may also exhibit some cytotoxic features. These and the remaining tests on PEEK composites, were carried out mainly for carbon fillings, which directly creates the need to conduct similar research over glass-reinforced PEEK (PEEK GRF) [26,27,28,29]. PEEK GRF with 30% of the reinforcement (PEEK GRF30) finds its use in i.a. modular implants for direct skeletal attachment of limb prosthesis, as it allows to reduce stress-shielding intensity due to relatively low, and comparable to bone’s, stiffness [30]. In the authors’ knowledge, there are no studies that would include the analyses of cells viability on PEEK GRF, characterized by pre-damaged surface, reflecting the damage that can be created due to the high stresses achieved in this process generated during the implantation of, e.g., press-fit implant.

In the case of the need of higher mechanical properties or individual adaptation of the implant’s shape to the patent’s anthropometric parameters, as often as plastic composites, methods of 3-dimensional printing with metallic alloys are in common use [23]. In this case, the most widely used is the selective laser melting (SLM) together with Ti6Al4V titanium alloy powder (due to the method used, it is often called Ti6Al4V SLM) [24].

Titanium and its alloys have an excellent tensile strength to density ratio and high resistance to corrosion and fatigue [31]. However, at the same time, literature sources report numerous technological problems [32,33]. The solution to the technological deficiencies of powder metallurgy is incremental techniques that allow us to generate complex surfaces and obtain materials of high purity [24]. What is more, the generative SLM technology allows us to create implants with high geometric complexity and properly shaped biomechanical properties corresponding to the characteristics of bone tissue, as well as porosity and roughness enabling cell proliferation and implant osseointegration with the surrounding tissue [34,35].

Literature reports show that in recent years a large group of patients has been successfully treated with the use of custom-made implants that were manufactured using SLM [36,37,38]. As these reports are relatively recent, little is still known about the biocompatibility of metallic implants produced by additive technologies. However, it is well-known knowledge that implant topography influences osteoblast proliferation and differentiation, hence the conclusion that surface modification of titanium implants can improve or deteriorate osteoblastic cell–implant interaction [39]. This means that there is a need to carry out appropriate tests clearly confirming the effectiveness of SLM as a technique for producing implants with Ti6Al4V SLM.

Analyzing the above-mentioned materials simultaneously with the use of the same methods would create a possibility not only to determine their actual biofunctionality but also to objectively compare metal and composite polymer as well as manufacturing techniques, which are often used for implants designated for the same purpose (implants for direct skeletal attachment of limb prosthesis, spinal implants, etc.). Currently, the literature is usually limited to analyses of metallic or composite material, omitting simultaneous research on described materials, providing the results that have to be interpreted and which cannot be directly compared due to the evaluation of different variables or the use of different methods. Moreover, there is no comprehensive research that approximates the influence of surface topography (considering various roughness parameters such as Ra, Rp, Rv or Rz) of materials described in this section (i.e., PEEK GRF30 and Ti6Al4V SLM) on viability of human osteoblasts, which was the aim of presented article.

## 2. Materials and Methods

### 2.1. Preparation of Samples and Modifications of Their Surface

In order to conduct the research, nine samples of PEEK GRF30, Ti6Al4V SLM as well as forged Ti6Al4V were prepared. The samples were cylindrical with a diameter of 10 mm and height of 5 mm. PEEK GRF30 samples were manufactured by injection molding, Ti6Al4V SLM samples were created with consideration of appropriate laser beam’s parameters (diameter = 0.10 mm, power = 190 W, speed = 500 mm/s, energy density = 127 J/mm^3^, porosity < 0.5%) and thickness layer of 0.03 mm with subsequent recrystallization annealing (850 °C for two hours), while Ti6Al4V by forging (at 900 °C) also with subsequent recrystallization annealing (800 °C for one hour). The front (tested) surfaces of the samples were machined first with sandpaper using its gradation from 120 to 2000.

Subsequently, front faces of eight samples of each material (leaving one polished) were mechanically damaged by their single and manual linear displacement of 100 mm sandpaper surface. Constant pressure was maintained during the movement of the sample. Each of eight samples was treated with sandpaper of different gradation: 240, 400, 600, 800, 1000, 1200, 1500 and 2000. The obtained surface roughness reflects the possibility of implant damage during i.a. its pressing into reamed medullary cavity, that generates high stresses in the process, as was indicated in the one of the authors recent paper [40].

**Figure 1:**
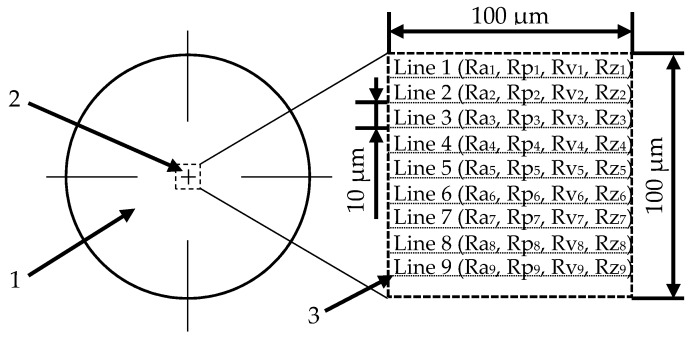
The method of applying profile lines to determine the average roughness of the tested surface: 1—tested sample, 2—analyzed area and 3—profile line.

The surfaces were then examined using a confocal microscope (LEXT OLS4000, Olympus Tokyo, Japan) to determine the effect of the previously performed modification in the surfaces roughness. In the microscope’s software, nine profile lines with a length of 100 μm were applied to the central part of the sample at fixed distances of 10 μm (Figure 1). For the profiles obtained, the individual surface roughness was determined (Ra_1–9_, Rp_1–9_, Rv_1–9_ and Rz_1–9_). Afterwards, their values were averaged to approximate the overall surface roughness for the tested sample.

### 2.2. Evaluation of Primary Human Osteoblasts with the Use of MTT Test

Primary human osteoblasts (Human Osteoblasts, PromoCell, Heidelberg, Germany), isolated from cancellous bone of femoral head, were used in the study. The conducted tests for the presence of bacteria (including mycoplasma) and fungi (including yeast) in cells were negative. Furthermore, no HIV-1 and hepatitis B or C virus were detected in the cells’ donor. The cells were from the second passage. Cytotoxicity tests were performed in accordance with ISO 10993-5:2009 [41].

Cells were subcultured in an incubator (Incubator Galaxy 170R(S) HTD, Eppendorf AG, Wesseliing-Berzdorf, Germany), accordingly to the manufacturer’s instructions (at 37 °C, in an atmosphere containing 5% CO_2_ and 95% humidity; the medium was changed after one day). Obtaining normal growth of the culture enabled the transfer of the part of the cells to a vessel containing fresh medium (so-called passaging). The passage was as follows: the cell suspension was washed several times with warm PBS solution. The cells were then separated from the vessel walls using a DetachKit with a pH of 7.4—a solution containing 0.04% trypsin and 0.03% versenic acid. Then, a medium containing an additional trypsin inhibitor was added to the tube. The resulting solution was transferred to sterile tubes, placed in a centrifuge (1200 rpm) and centrifuged for 3 min at room temperature to separate the cells from the solution. Viable cells were resuspended in medium (changed every two days) and transferred to more culture vessels. The passaging was carried out several times after the cells reached full confluence (degree of coverage of the culture vessel surface).

On the sixteenth day after the start of culture (passage 6/7/8), cells were seeded on the surface of the samples placed in a 24-well plate. The samples were sterilized before starting their cultivation. The risk of microbial contamination was reduced by briefly immersing the samples in 70% isopropanol and rinsing with deionized water, followed by steam sterilization at 121 °C for 20 min (Systec VX-VE, Systec GmbH Labor-Systemtechnik, Linden, Germany)—it provides oxide film facilitating cells to attach to the implant surface and not causing inflammatory reactions in long-term in vivo tests. After sterilization process, the samples were transferred to a 24-well cell culture plate.

Then, osteoblasts, in a density of 50,000 per well, were cultured on samples of analyzed materials as well as on the bottom of wells to obtain control samples. The number of osteoblasts and their viability was determined using an automated cell counter (EVE, NanoEnTek, Seoul, Korea), which allows us to count cells as well as distinguish live cells from dead ones. The counter’s detection system is based on staining cells with trypan blue. As only dead cells are stained, it is possible to reject their number from the total number of cells. This allowed us to prepare suspensions containing 50,000 live osteoblast cells that were cultured on the surfaces of tested samples. After culturing, 1 mL of growth medium, optimized for in-vitro culturing of osteoblasts used (Osteoblast Growth Medium, PromoCell), was added to each well.

Accordingly to the standard, the samples were placed between two negative controls (NC). NC is a non-toxic control that involves cells growing on the surface of a well. Empty wells were filled with phosphate buffered saline (PBS) to maintain uniform evaporation of liquids from the plate. Cell growth lasted for 48 h. After first 24 h, a positive control (PC) of 0.2% Triton X-100, was added. The 0.2% solution was prepared from 100% one by a series of dilutions using PBS firstly to obtain 10% and then to obtain 1% solution. Subsequently, 0.2 mL of a 1% triton solution was added to 0.8 mL of growth medium in wells of the plate, which were designated as PC. The triton is a lysing reagent, i.e., destroying cell membranes, which causes a significant decrease in viability of osteoblasts that can be considered as cytotoxic property [42]. After 48 h of culturing osteoblasts on samples, the MTT test was carried out on the basis of the cells manufacturer instructions. Formazan crystals precipitated by the reduction of the tetrazolium salt were dissolved using a 70% solution of isopropanol with HCl at a concentration of 0.04 mol. Cells viability was determined as a percentage value related to the average results obtained for NC (Equation (1)). Prior to the viability calculations, 100 μL of the obtained dissolved formazan crystals were collected from each well and transferred to a 96-well plate. This procedure was repeated six times. Finally, the obtained values were averaged. As a threshold value, below which the material was considered as cytotoxic, 70% of the value obtained for the NC was taken.

Cells viability (%) = (A_O_/A_NC_) × 100%, (1)

where: A_O_—absorbance of osteoblasts and (A_NC_)—average absorbance of all negative controls on the left and right side of the plate.

The absorbance of osteoblasts was measured using a plate reader (VICTOR X4, Perkin Elmer, Waltham, MA, USA), for λ of 570 nm and 650 nm (determined on the basis of the ISO standard). The cells studied are characterized by a partial absorption of the electromagnetic wavelength of 570 nm, while they are permeable to a wavelength of 650 nm that is only partially absorbed by the background, which also partially absorbs a wavelength of 570 nm. To calculate the absorbance of osteoblasts, the absorbance obtained for λ = 570 nm was subtracted from the absorbance obtained when using λ = 650 nm (Equation (2)).

A_O_ = (∑^n=6 I=1^ (A_570n_ − A_650n_))/n, (2)

where: A570—absorbance at λ = 570 nm, A650—absorbance at λ = 650 nm.

Similarly, the parameter (A_NC_) was measured (Equation (3)).

A_NC_ = 0.5 ((∑^n=6 I=1^ (A_NCL570n_ − A_NCL650n_))/n) + (∑^n=6 I=1^ (A_NCR570n_ − A_NCR650n_)/n), (3)

where: A_NCL570_—absorbance of the NC placed on the left side of the plate for λ = 570 nm, A_NCR650_—absorbance of the NC placed on the right side of the plate for λ = 650 nm.

For comparative purposes, the mean absorbance of the PC was also determined (Equation (4)).

**Figure 2:**
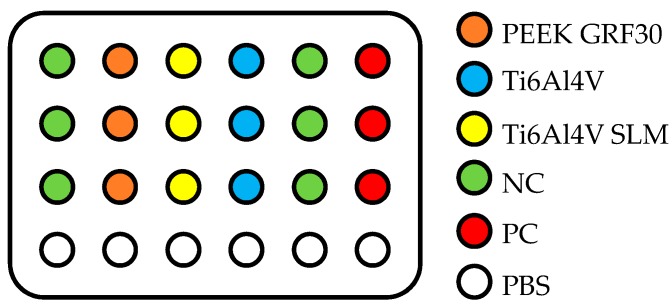
Exemplary arrangement of the samples during the MTT test.

A_PC570_ = (∑^n=6 i=1^ (A_PC570n_ − A_PC650n_))/n, (4)

where: A_PC570_—absorbance of the PC for λ = 570 nm and A_PC650_—absorbance of the PC for λ = 650 nm. Due to spatial limitations, the tests were carried out in three series, six samples in each, using the 6th, 7th and 8th passage of the cells sequentially. In-vitro tests were carried out six times in each series. The obtained absorbance results were consistently referred to the calculated parameters obtained in each series. A schematic diagram of an exemplary arrangement of samples in a single series is presented in Figure 2.

Finally, the obtained averaged results characterizing the tested materials were statistically processed using a one-way ANOVA and the Tukey’s test for multiple comparisons (i.e., a test of significant differences between averaged values), and values for which a *p* value <0.05 was assumed as statistically significant.

## 3. Results and Discussion

### 3.1. The Influence of Mechanical Treatment on Surface Roughness

The exemplary surfaces of PEEK GRF30, Ti6Al4V SLM and Ti6Al4V obtained after mechanical treatment with sandpaper are presented respectively in Figure 3, Figure 4 and Figure 5. Enlarged areas chosen for determining surfaces roughness are presented in Supplementary (Figures S1–S3). Each image was obtained with the use of confocal microscope during the measurements of surface roughness. Moreover, the surface topography obtained for previously presented samples is presented in Supplementary, again respectively for PEEK GRF30 (Figures S4–S12), Ti6Al4V SLM (Figures S13–S21) and Ti6Al4V (Figures S22–S30).

**Figure 6:**
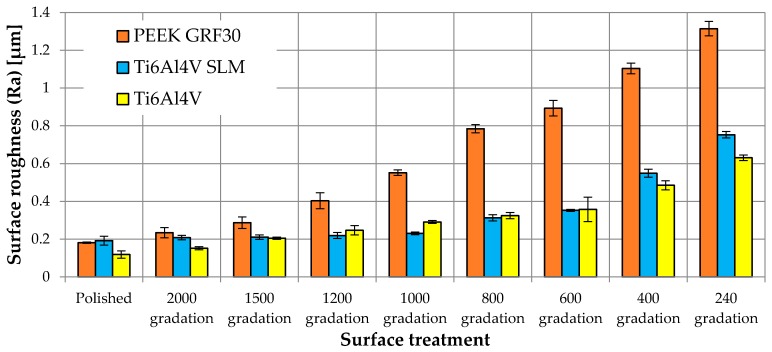
Surface roughness for individual methods of surface preparation.

The influence of mechanical treatment on the surface roughness of samples of tested materials is presented in Figure 6.

The first observable feature, before analyzing the results of the MTT test, is the variable influence of the mechanical treatment with sandpaper of various gradations on the surface roughness of PEEK GRF30 and Ti6Al4V SLM as well as Ti6Al4V. With the use of the same paper gradation or the same polishing process, different surface roughness were obtained for both tested materials. The roughness obtained for PEEK GRF30 was in some cases even twice as high as the roughness obtained for Ti6Al4V SLM or Ti6Al4V, e.g., after treatment with sandpaper of 240 gradation the roughnesses values (Ra) were 1.31 μm, 0.75 μm and 0.63 μm for PEEK GRF30, Ti6Al4V SLM and Ti6Al4V respectively. The differences can be caused by the pulling out the glass fibers located on the surface of PEEK GRF30. It results in creating visible microholes that influence the surface roughness. Nevertheless, in some cases, similar surface roughness values were obtained (although by treatment with paper of different gradations), which allowed further and objective comparison of cells viability for a similar surface condition of the materials tested. Moreover, a small standard deviation suggests a small spread in the results, indicating a relatively regular surface. The obtained values for the polishing treatment are close to the values reported in the literature (Ra = 0.1 μm), which means that the surfaces used in analyses were prepared correctly [43,44].

### 3.2. Determination of Osteoblasts Viability Using MTT Test

**Figure 7:**
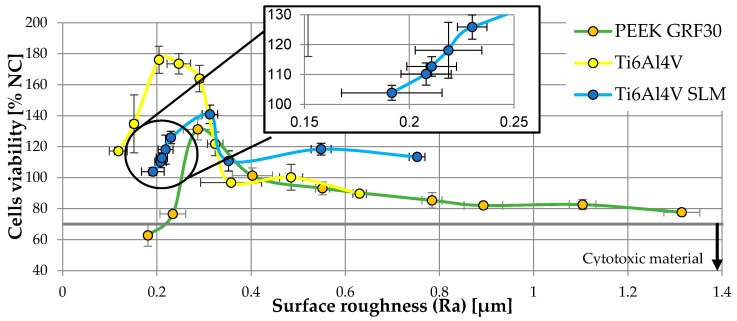
Viability of osteoblasts in relation to the roughness of tested materials.

Figure 7 shows the viability of osteoblasts in relation to the roughness of tested materials.

Table 1 presents all the obtained values of the remaining parameters clearly defining the topography of the obtained surfaces to clearly present their influence on osteoblasts viability.

**Figure 8:**
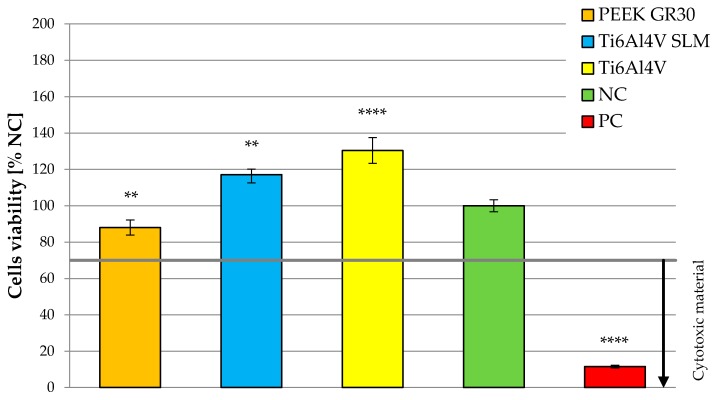
Averaged value of cells viability on tested materials (** significance to negative control (NC)—for *p* < 0.01 and **** significance to NC—for *p* < 0.0001).

Finally, Figure 8 presents the average viability of cells on the tested materials to the overall evaluation of their cytotoxicity.

When analyzing results presented in current subsection, a significant difference between the cells viability of the tested materials is observable. For surface roughness of Ra ≤ 0.3 μm, cells viability on Ti6Al4V was nearly twice as high as in the case of PEEK GRF30. For Ra > 0.3 μm, cells viability on both materials was similar. It can also be noted that the optimal roughness, in the case of the materials tested, was approx. 0.2–0.3 μm (maximum cells viability was 176.03%, for Ti6Al4V with Ra = 0.2 μm while the highest viability of 131.15% for PEEK GRF30 was with of Ra = 0.29 μm). What is more, further analysis can also indicate that for polished PEEK GRF30 sample (Ra = 0.18 μm), cell viability was below 70% (in this case the viability was 62.72%). This suggests the lack of sufficiently high biocompatibility and the need to change the surface condition of polished PEEK GRF30.

When analyzing the results for Ti6Al4V SLM, it can be noticed that, as in the case of two other materials, the viability of osteoblasts rises with the increase of roughness from the one obtained during the polishing process. It should be taken into account that surface state characterized by Ra = 0.30 μm also allowed to achieve the highest possible cells viability. Again, with further increase in surface roughness, osteoblasts viability was decreasing however, it was maintained on higher level in relation to PEEK GRF 30 and Ti6Al4V. It can be caused by the presence of microholes formed in the process of 3D printing, which most likely improve the surface topography in terms of cells viability.

The results suggest that there may exist a certain surface condition, different from polished, on which osteoblast cells are characterized by a higher viability. This statement is confirmed in the available literature, according to which the Ra value of 0.3 μm, is optimal for achieving adequate cell viability [45]. This is also confirmed by other studies, which state that external cracks on surfaces can positively affect the viability of osteoblasts [46].

The results obtained also indicate that the cell viability was small for significant roughness, i.e., in this case resulting from the machining outer surface of the material with the low gradation sandpaper. Surface damage may occur during the implantation of press-fit implant (due to the high stresses) like an implant for direct skeletal attachment of limb prosthesis. It can result in reduction of its biocompatibility and consequently lowered possibility of obtaining appropriate primary stabilization. This would explain the reason for removal of ITAP implants from individual cases of its use in dogs after limb amputation due to osteointegration failure [20].

A similar effect on the cells viability can be noticed while analyzing the influence of three more parameters describing surface topography (i.e., Rp, Rv and Rz). On their basis it can be also concluded that the roughness slightly higher than the one obtained after polishing, allows us to increase osteoblasts viability. This confirms previously presented conclusions determined on the dependency presented in Figure 8.

### 3.3. Future Work

In the future the authors plan to conduct the evaluation of cytotoxicity of PEEK GRF30 and Ti6Al4V SLM with consideration of different topographies, which were considered in the presented study, as well as to use more samples in order to obtain more comprehensive research. Different topographies could be created via mechanical treatment, carried out in more than one direction to create, e.g., bidirectional, circumferential or random damage in samples surface. This could provide a better understanding of the influence of various roughness on human osteoblasts viability. Moreover, the authors also consider examining the effect of wear particles on the cells response as some studies suggest that above-mentioned debris may have a potential role in bone resorption around orthopaedic implants.

## 4. Conclusions

Cells viability studies are basic research conducted to evaluate biocompatibility of the implant, allowing us to estimate the tissue response after its implantation. The study with the use of MTT test, presented in the paper, allowed the initial evaluation of functionality of PEEK GRF30, Ti6Al4V SLM and Ti6Al4V in post-implantation conditions in typical polished state or after surface damage in longitudinal direction.

The general results indicate that the biocompatibility requirements were fulfilled by all of the tested materials. However, when selecting appropriate material for implants, surface roughness should be taken into account, which could significantly influence the viability of human osteoblasts.

## Figures and Tables

**Figure 3:**
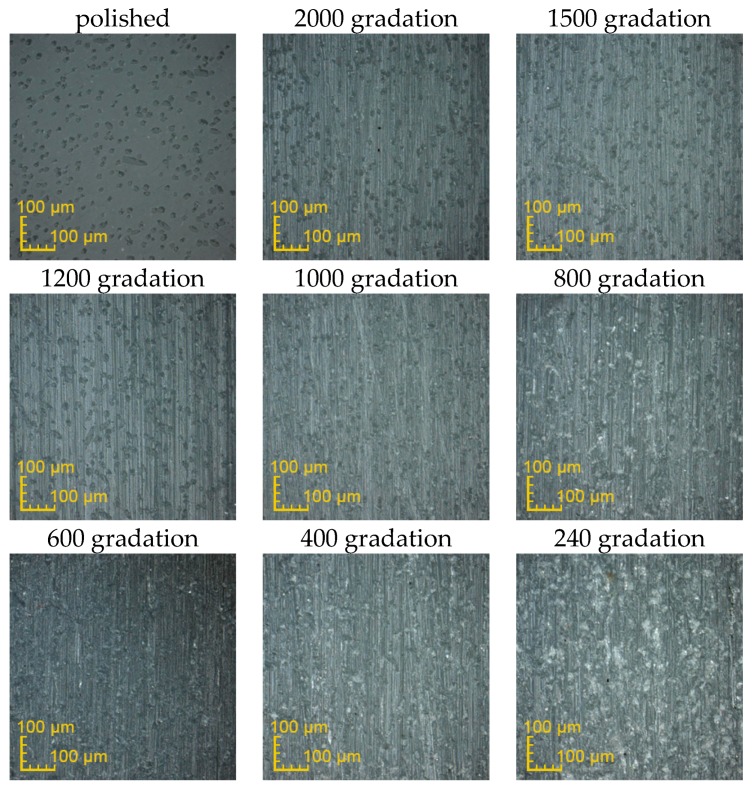
Surface condition of PEEK GRF30 samples obtained after treating it with the sandpaper of various gradations (magnification ×426).

**Figure 4:**
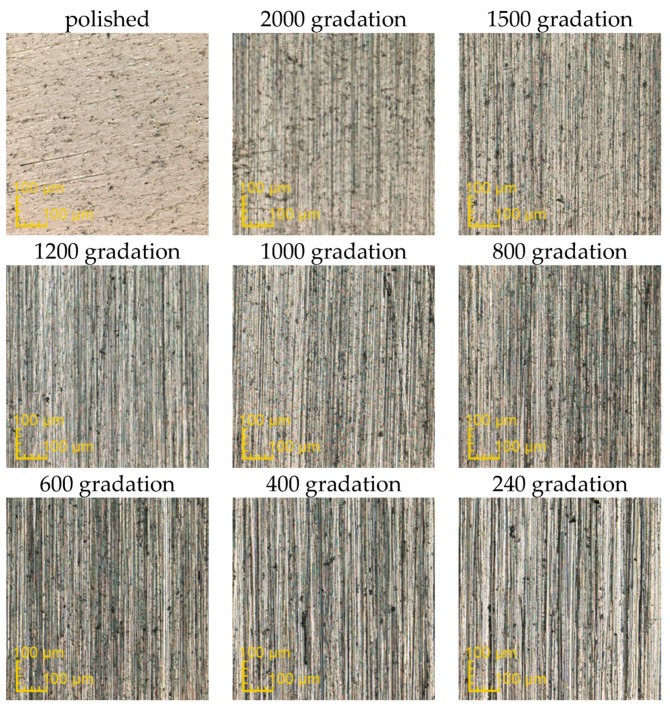
Surface condition of Ti6Al4V selective laser melting (SLM) samples obtained after treating it with the sandpaper of various gradations (magnification ×426).

**Figure 5:**
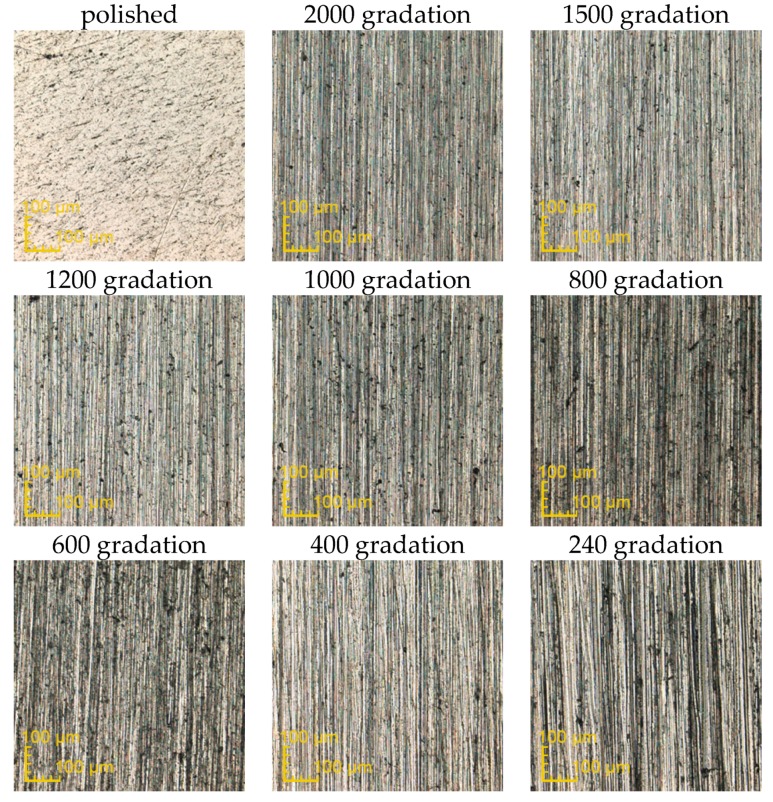
Surface condition of Ti6Al4V samples obtained after treating it with the sandpaper of various gradations (magnification ×426).

**Table 1:** Surface roughness parameters (Ra, Rp, Rv and Rz) and their influence on cells viability.

Material	Surface Treatment	Surface Roughness Parameter								Cells Viability (% NC)	
		Ra (µm)		Rp (µm)		Rv (µm)		Rz (µm)			
		Mean	SD	Mean	SD	Mean	SD	Mean	SD	Mean	SD
PEEK GRF30	Polished	0.181	0.004	0.630	0.055	1.040	0.170	1.669	0.124	62.72	7.07
	2000 gradation	0.234	0.027	0.899	0.084	1.737	0.272	2.736	0.419	76.54	2.07
	1500 gradation	0.287	0.030	1.027	0.097	2.384	0.186	3.327	0.399	131.15	6.81
	1200 gradation	0.403	0.043	1.198	0.099	2.765	0.329	4.132	0.688	101.26	4.91
	1000 gradation	0.552	0.015	1.463	0.341	2.962	0.592	4.213	0.265	93.08	4.16
	800 gradation	0.784	0.022	2.594	0.488	3.104	0.610	5.850	1.045	85.31	4.96
	600 gradation	0.893	0.041	3.074	0.390	3.922	0.571	6.642	0.696	82.03	1.78
	400 gradation	1.104	0.028	3.844	0.356	4.382	0.471	8.256	0.144	82.58	3.30
	240 gradation	1.315	0.038	9.353	0.400	7.750	0.543	16.569	1.319	77.64	2.19
Ti6Al4V SLM	Polished	0.192	0.024	0.558	0.091	0.789	0.101	1.347	0.166	103.86	2.49
	2000 gradation	0.208	0.012	0.629	0.070	0.793	0.184	1.423	0.234	110.16	3.71
	1500 gradation	0.211	0.012	0.704	0.057	0.831	0.088	1.502	0.155	112.67	3.25
	1200 gradation	0.219	0.016	0.953	0.200	0.906	0.083	1.825	0.208	118.07	9.35
	1000 gradation	0.230	0.007	1.116	0.225	1.217	0.140	2.437	0.215	125.87	4.05
	800 gradation	0.313	0.017	1.221	0.076	1.541	0.163	2.820	0.362	140.85	5.92
	600 gradation	0.352	0.006	1.579	0.252	2.225	0.107	3.464	0.528	110.76	6.48
	400 gradation	0.549	0.021	2.232	0.603	2.681	0.479	4.656	0.851	118.36	3.84
	240 gradation	0.753	0.017	2.469	0.701	3.238	0.630	5.707	0.361	113.35	1.50
Ti6Al4V	Polished	0.118	0.019	0.512	0.107	0.531	0.031	1.105	0.034	117.11	2.28
	2000 gradation	0.152	0.008	0.575	0.018	0.651	0.156	1.162	0.191	134.70	18.65
	1500 gradation	0.205	0.006	0.582	0.073	0.749	0.078	1.330	0.022	176.03	8.76
	1200 gradation	0.247	0.025	0.755	0.083	1.046	0.070	1.801	0.126	173.51	6.62
	1000 gradation	0.291	0.008	0.972	0.281	1.071	0.201	2.163	0.057	163.87	8.53
	800 gradation	0.324	0.017	1.092	0.148	1.510	0.200	2.482	0.441	121.79	7.72
	600 gradation	0.358	0.065	1.275	0.179	1.629	0.111	3.028	0.678	96.76	1.63
	400 gradation	0.485	0.025	1.659	0.343	1.952	0.398	3.288	0.409	100.19	8.30
	240 gradation	0.631	0.014	2.582	0.237	3.390	0.419	5.972	0.318	89.67	1.59

## Supplementary Materials

The following are available online at https://www.mdpi.com/1996-1944/12/24/4189/s1, Figure S1: Enlarged areas of PEEK GRF30 samples, chosen for determining surfaces roughness (100 µm × 100 µm), Figure S2: Enlarged areas of Ti6Al4V SLM samples, chosen for determining surfaces roughness (100 µm × 100 µm), Figure S3: Enlarged areas of Ti6Al4V samples, chosen for determining surfaces roughness (100 µm × 100 µm), Figure S4: Surface topography of PEEK GRF30 sample obtained after treating it with the polishing cloth (magnification ×426), Figure S5: Surface topography of PEEK GRF30 sample obtained after treating it with the sandpaper of 2000 gradation (magnification ×426), Figure S6: Surface topography of PEEK GRF30 sample obtained after treating it with the sandpaper of 1500 gradation (magnification ×426), Figure S7: Surface topography of PEEK GRF30 sample obtained after treating it with the sandpaper of 1200 gradation (magnification ×426), Figure S8: Surface topography of PEEK GRF30 sample obtained after treating it with the sandpaper of 1000 gradation (magnification ×426), Figure S9: Surface topography of PEEK GRF30 sample obtained after treating it with the sandpaper of 800 gradation (magnification ×426), Figure S10: Surface topography of PEEK GRF30 sample obtained after treating it with the sandpaper of 600 gradation (magnification ×426), Figure S11: Surface topography of PEEK GRF30 sample obtained after treating it with the sandpaper of 400 gradation (magnification ×426), Figure S12: Surface topography of PEEK GRF30 sample obtained after treating it with the sandpaper of 240 gradation (magnification ×426), Figure S13: Surface topography of Ti6Al4V SLM sample obtained after treating it with the polishing cloth (magnification ×426), Figure S14: Surface topography of Ti6Al4V SLM sample obtained after treating it with the sandpaper of 2000 gradation (magnification ×426), Figure S15: Surface topography of Ti6Al4V SLM sample obtained after treating it with the sandpaper of 1500 gradation (magnification ×426), Figure S16: Surface topography of Ti6Al4V SLM sample obtained after treating it with the sandpaper of 1200 gradation (magnification ×426), Figure S17: Surface topography of Ti6Al4V SLM sample obtained after treating it with the sandpaper of 1000 gradation (magnification ×426), Figure S18: Surface topography of Ti6Al4V SLM sample obtained after treating it with the sandpaper of 800 gradation (magnification ×426), Figure S19: Surface topography of Ti6Al4V SLM sample obtained after treating it with the sandpaper of 600 gradation (magnification ×426), Figure S20: Surface topography of Ti6Al4V SLM sample obtained after treating it with the sandpaper of 400 gradation (magnification ×426), Figure S21: Surface topography of Ti6Al4V SLM sample obtained after treating it with the sandpaper of 240 gradation (magnification ×426), Figure S22: Surface topography of Ti6Al4V sample obtained after treating it with the polishing cloth (magnification ×426), Figure S23: Surface topography of Ti6Al4V sample obtained after treating it with the sandpaper of 2000 gradation (magnification ×426), Figure S24: Surface topography of Ti6Al4V sample obtained after treating it with the sandpaper of 1500 gradation (magnification ×426), Figure S25: Surface topography of Ti6Al4V sample obtained after treating it with the sandpaper of 1200 gradation (magnification ×426), Figure S26: Surface topography of Ti6Al4V sample obtained after treating it with the sandpaper of 1000 gradation (magnification ×426), Figure S27: Surface topography of Ti6Al4V sample obtained after treating it with the sandpaper of 800 gradation (magnification ×426), Figure S28: Surface topography of Ti6Al4V sample obtained after treating it with the sandpaper of 600 gradation (magnification ×426), Figure S29: Surface topography of Ti6Al4V sample obtained after treating it with the sandpaper of 400 gradation (magnification ×426), Figure S30: Surface topography of Ti6Al4V sample obtained after treating it with the sandpaper of 240 gradation (magnification ×426).
