# Supplementary material for: Influence of the Surface Roughness of PEEK GRF30 and Ti6Al4V SLM on the Viability of Primary Human Osteoblasts Determined by the MTT Test
Source: Materials (Basel). 2019 Dec 13;12(24):4189. doi: 10.3390/ma12244189 (PMC6947449; doi:10.3390/ma12244189)
Supplement: Supplementary file 1 [file materials-12-04189-s001.pdf]

Supplementary Materials: Influence of the Surface Roughness of PEEK GRF30 and Ti6Al4V SLM on the Viability of Primary Human Osteoblasts Determined by the MTT Test

Piotr Prochor and Żaneta Anna Mierzejewska

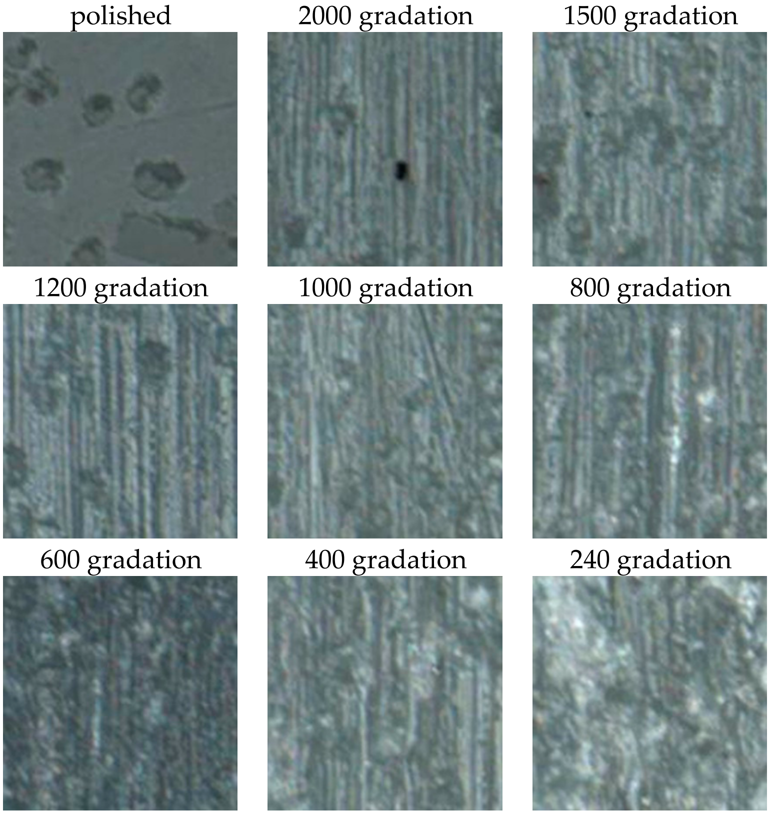

Figure S1. Enlarged areas of PEEK GRF30 samples, chosen for determining surfaces roughness (100 μm × 100 μm).

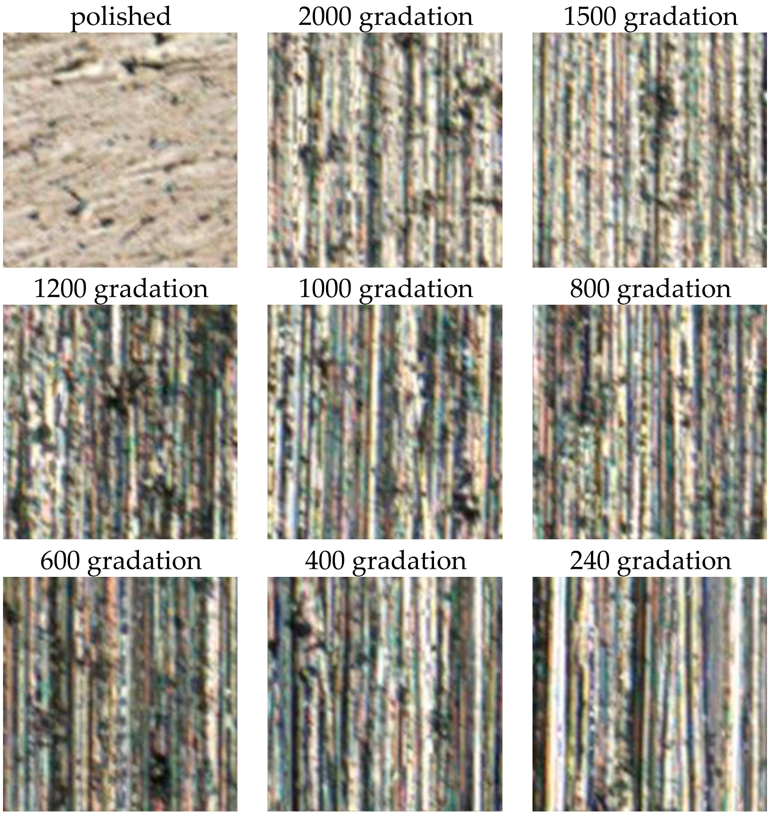

Figure S2. Enlarged areas of Ti6Al4V SLM samples, chosen for determining surfaces roughness (100 μm × 100 μm).

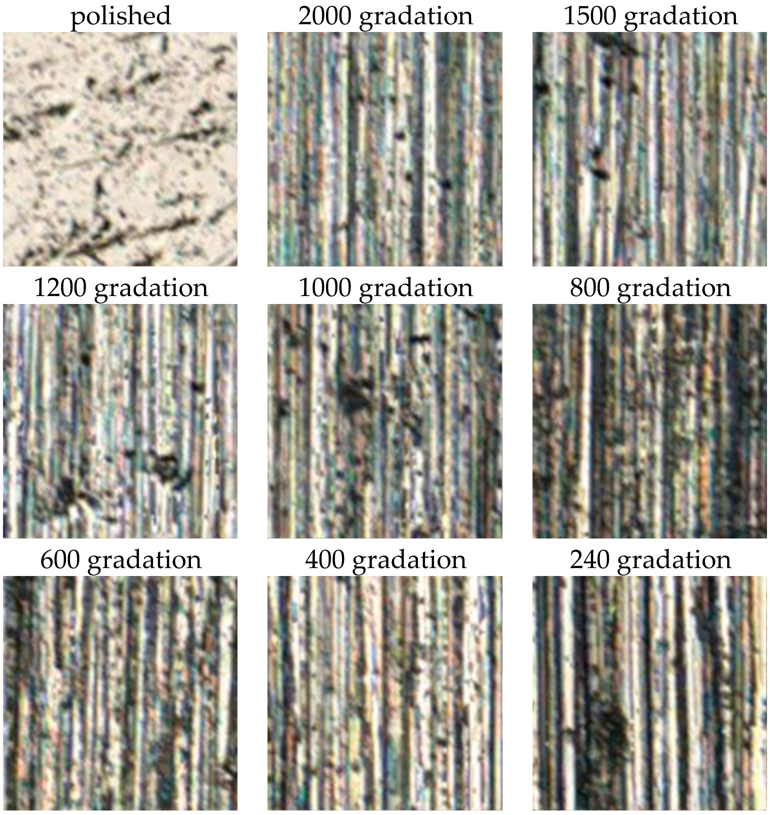

Figure S3. Enlarged areas of Ti6Al4V samples, chosen for determining surfaces roughness (100 μm × 100 μm).

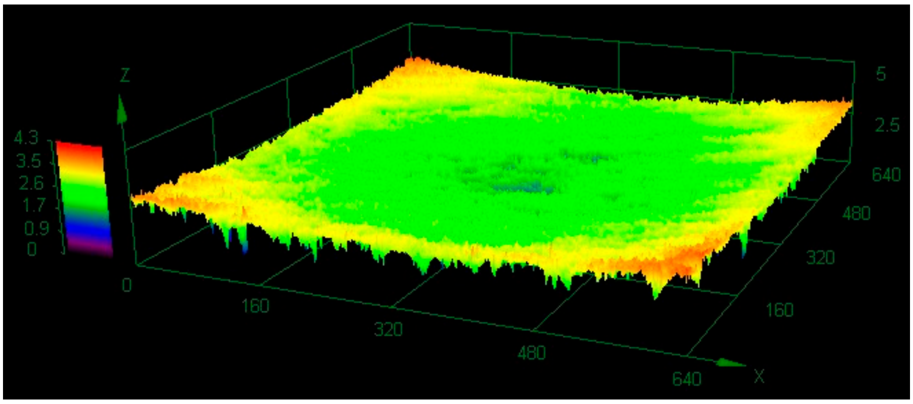

Figure S4. Surface topography of PEEK GRF30 sample obtained after treating it with the polishing cloth (magnification ×426).

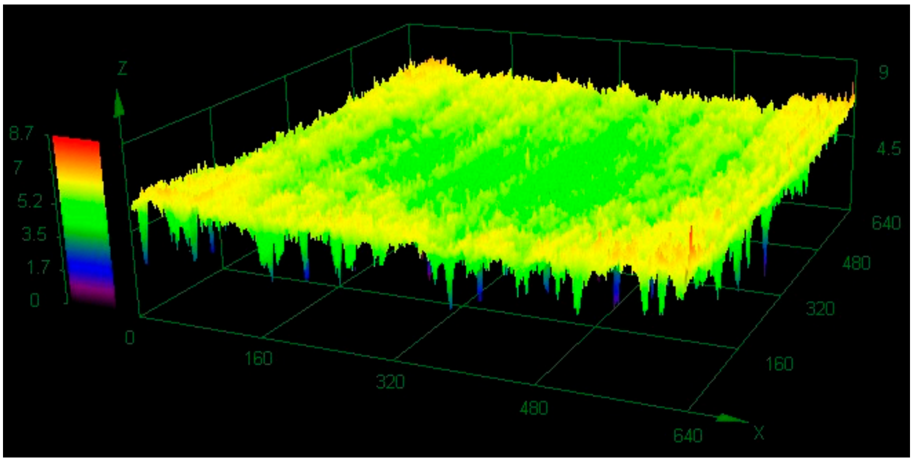

Figure S5. Surface topography of PEEK GRF30 sample obtained after treating it with the sandpaper of 2000 gradation (magnification ×426).

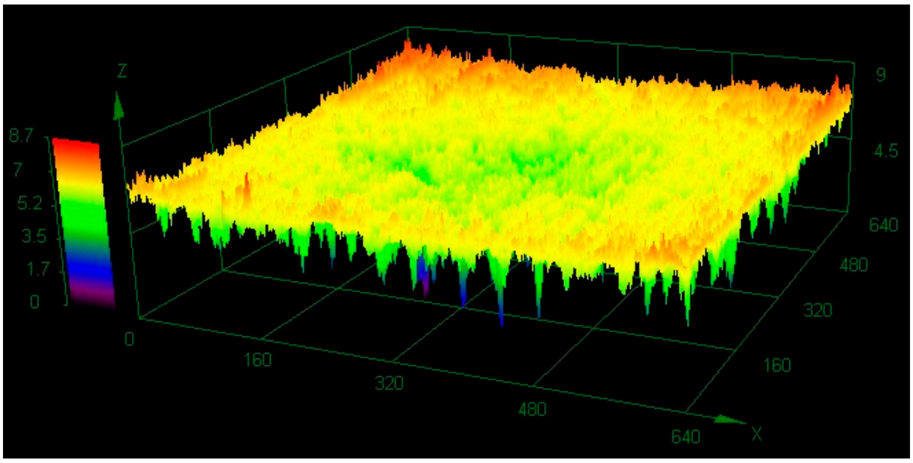

Figure S6. Surface topography of PEEK GRF30 sample obtained after treating it with the sandpaper of 1500 gradation (magnification ×426).

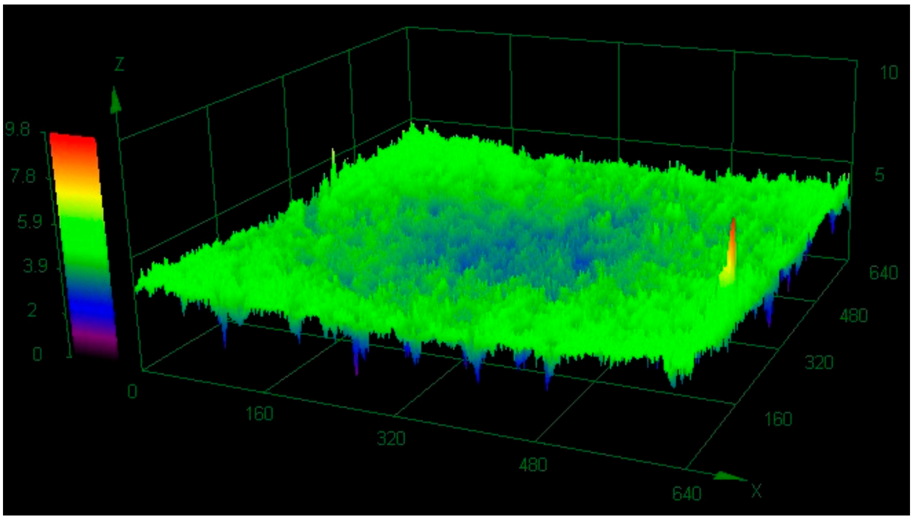

Figure S7. Surface topography of PEEK GRF30 sample obtained after treating it with the sandpaper of 1200 gradation (magnification ×426).

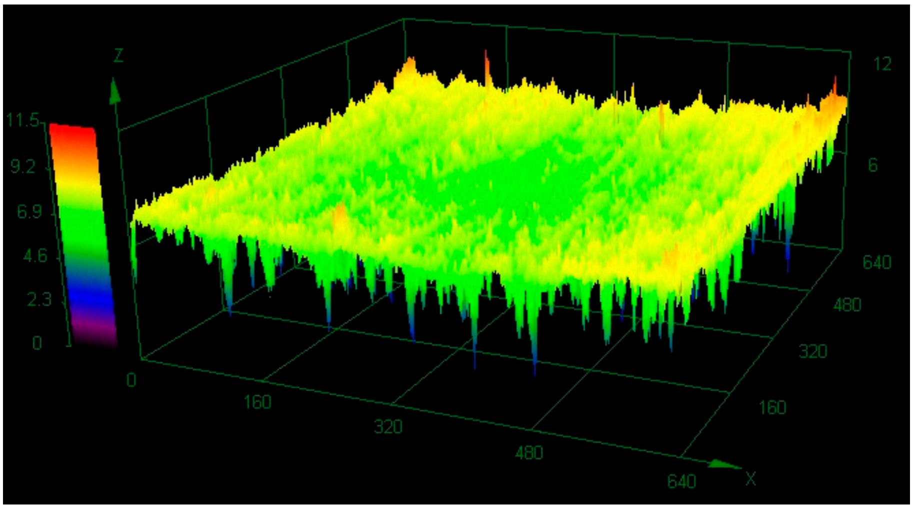

Figure S8. Surface topography of PEEK GRF30 sample obtained after treating it with the sandpaper of 1000 gradation (magnification ×426).

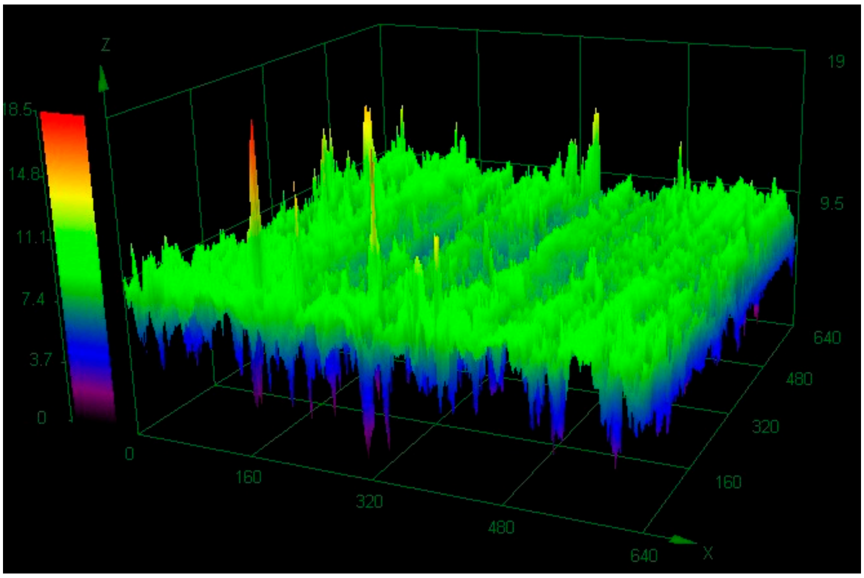

Figure S9. Surface topography of PEEK GRF30 sample obtained after treating it with the sandpaper of 800 gradation (magnification ×426).

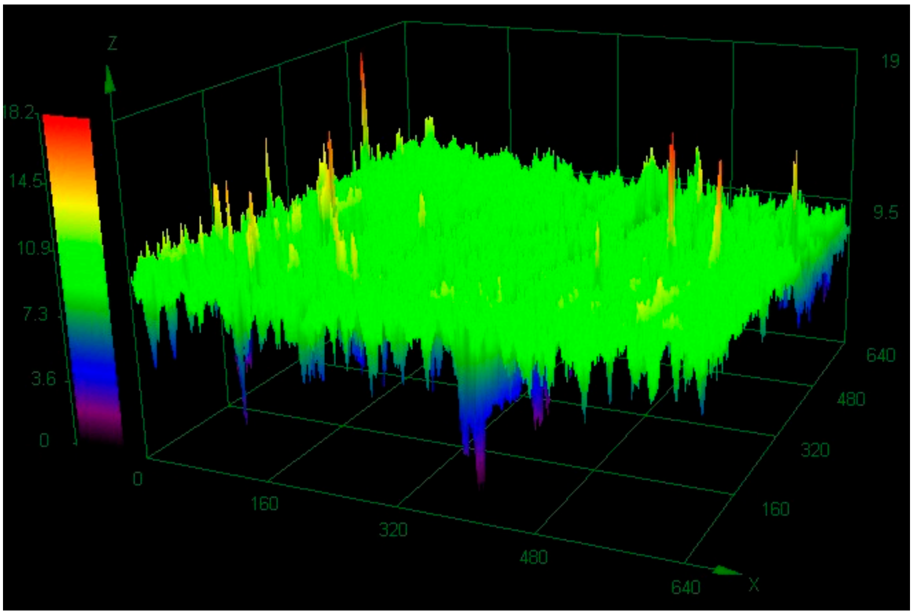

Figure S10. Surface topography of PEEK GRF30 sample obtained after treating it with the sandpaper of 600 gradation (magnification ×426).

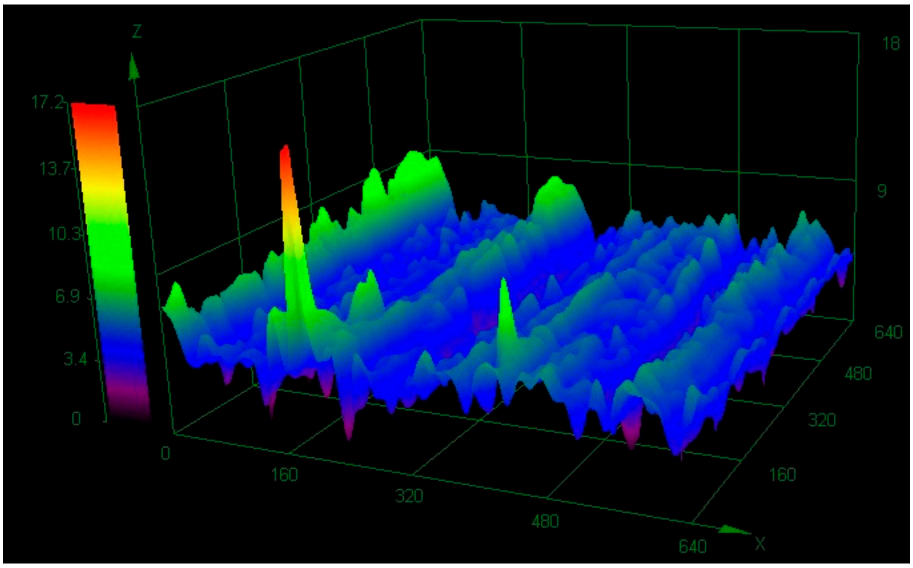

Figure S11. Surface topography of PEEK GRF30 sample obtained after treating it with the sandpaper of 400 gradation (magnification ×426).

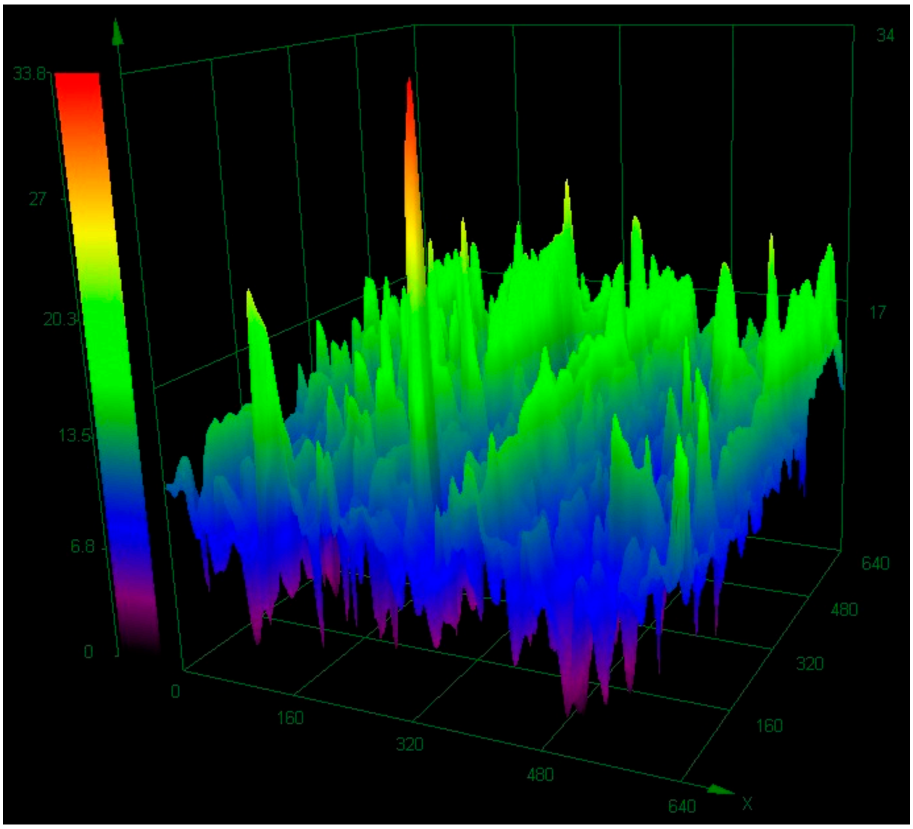

Figure S12. Surface topography of PEEK GRF30 sample obtained after treating it with the sandpaper of 240 gradation (magnification ×426).

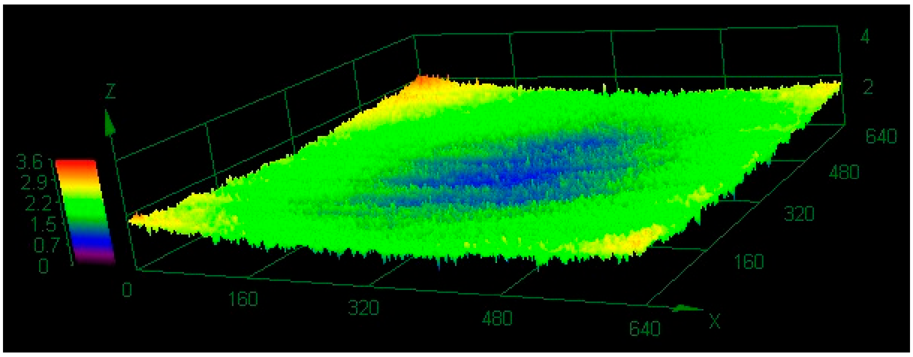

Figure S13. Surface topography of Ti6Al4V SLM sample obtained after treating it with the polishing cloth (magnification ×426).

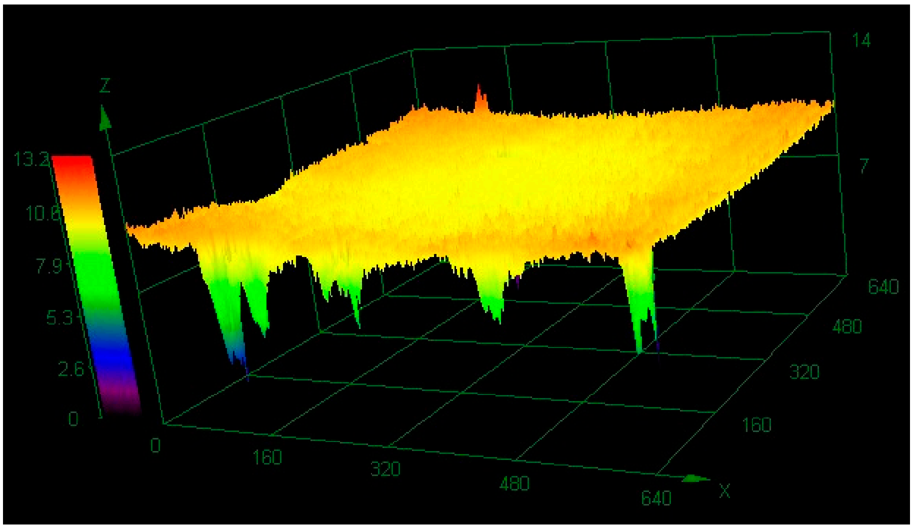

Figure S14. Surface topography of Ti6Al4V SLM sample obtained after treating it with the sandpaper of 2000 gradation (magnification ×426).

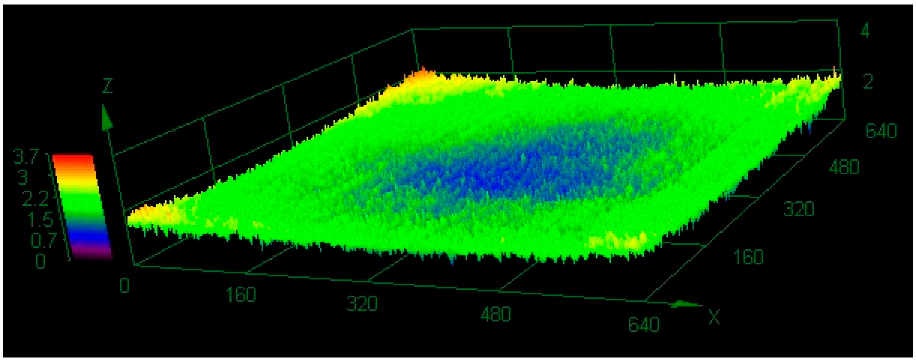

Figure S15. Surface topography of Ti6Al4V SLM sample obtained after treating it with the sandpaper of 1500 gradation (magnification ×426).

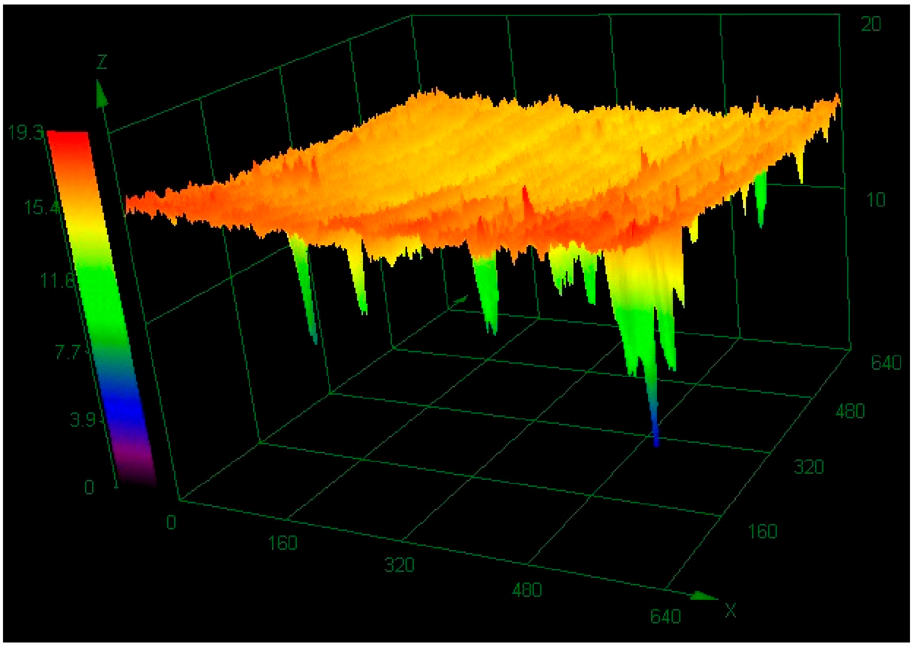

Figure S16. Surface topography of Ti6Al4V SLM sample obtained after treating it with the sandpaper of 1200 gradation (magnification ×426).

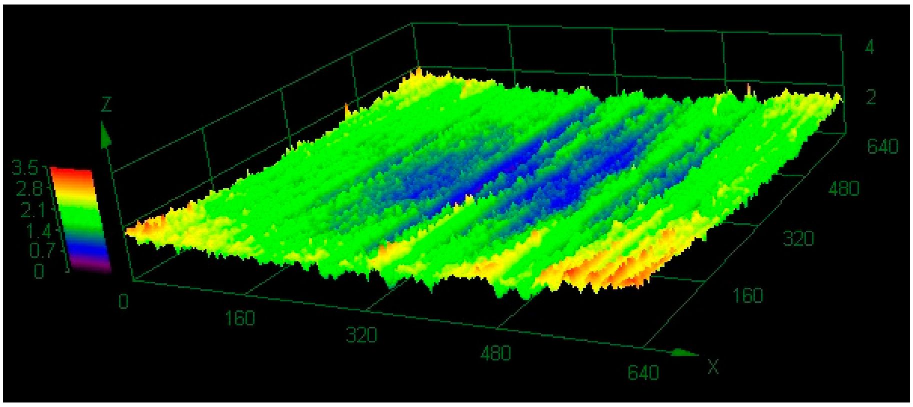

Figure S17. Surface topography of Ti6Al4V SLM sample obtained after treating it with the sandpaper of 1000 gradation (magnification ×426).

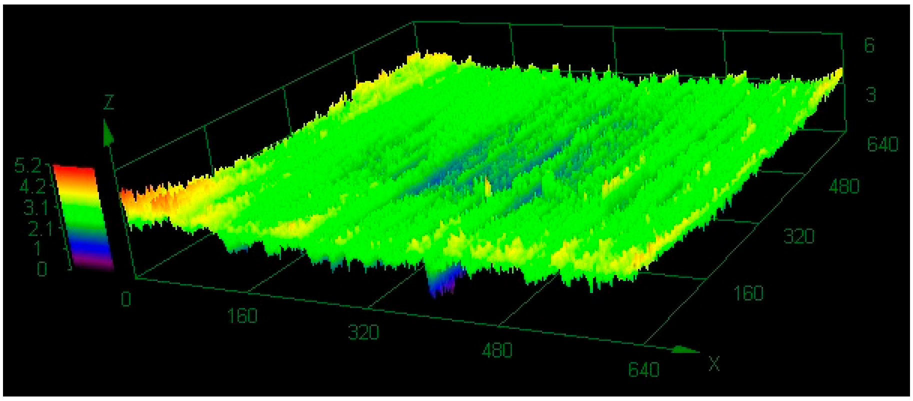

Figure S18. Surface topography of Ti6Al4V SLM sample obtained after treating it with the sandpaper of 800 gradation (magnification ×426).

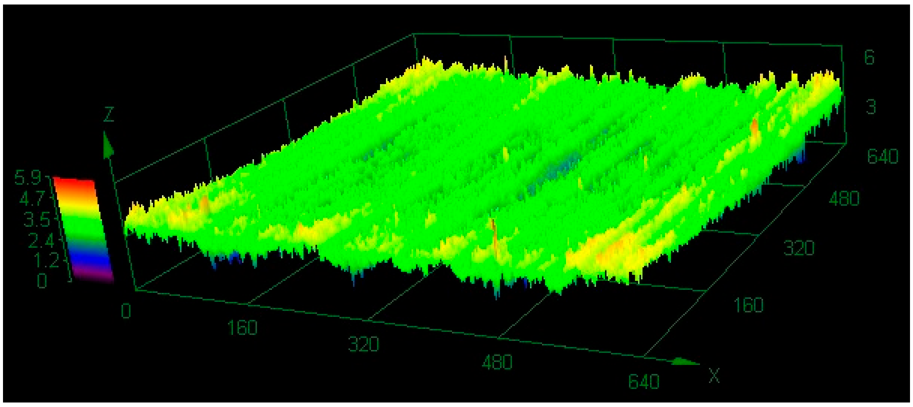

Figure S19. Surface topography of Ti6Al4V SLM sample obtained after treating it with the sandpaper of 600 gradation (magnification ×426).

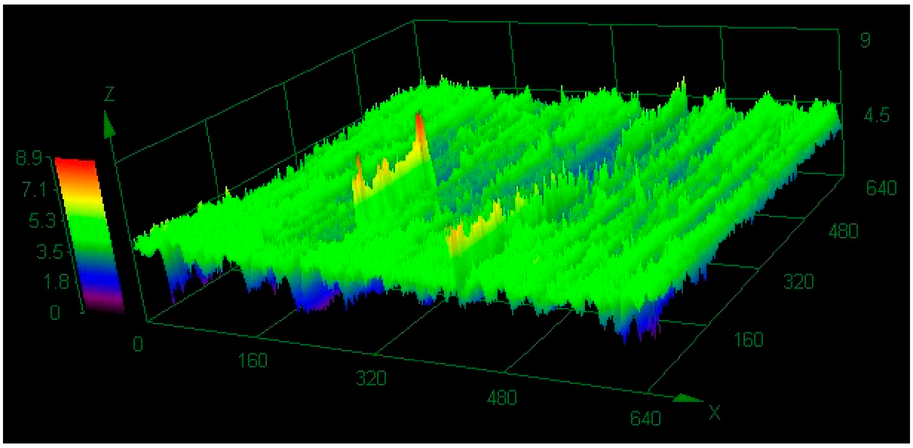

Figure S20. Surface topography of Ti6Al4V SLM sample obtained after treating it with the sandpaper of 400 gradation (magnification ×426).

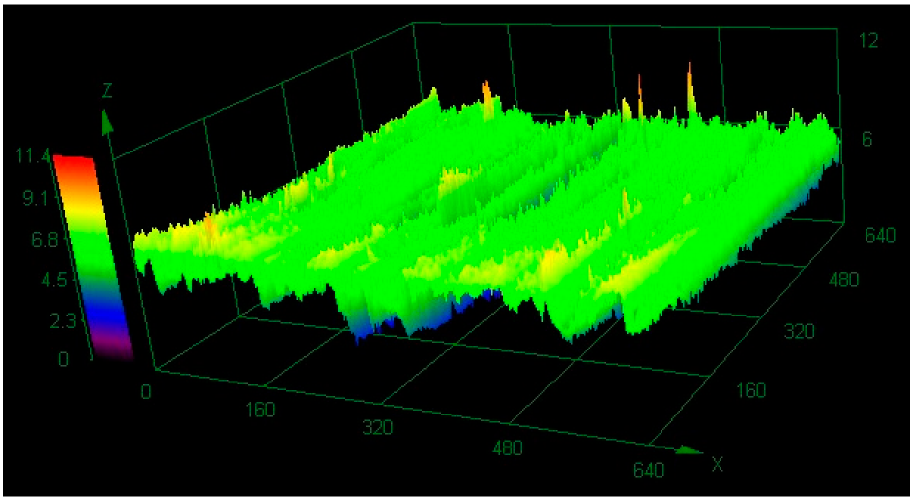

Figure S21. Surface topography of Ti6Al4V SLM sample obtained after treating it with the sandpaper of 240 gradation (magnification ×426).

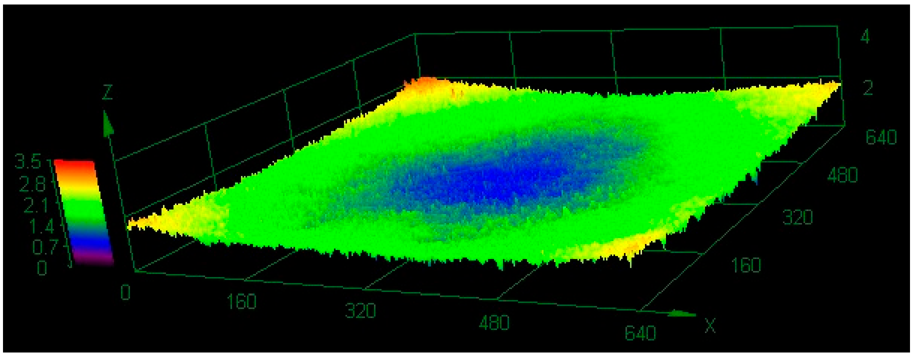

Figure S22. Surface topography of Ti6Al4V sample obtained after treating it with the polishing cloth (magnification ×426).

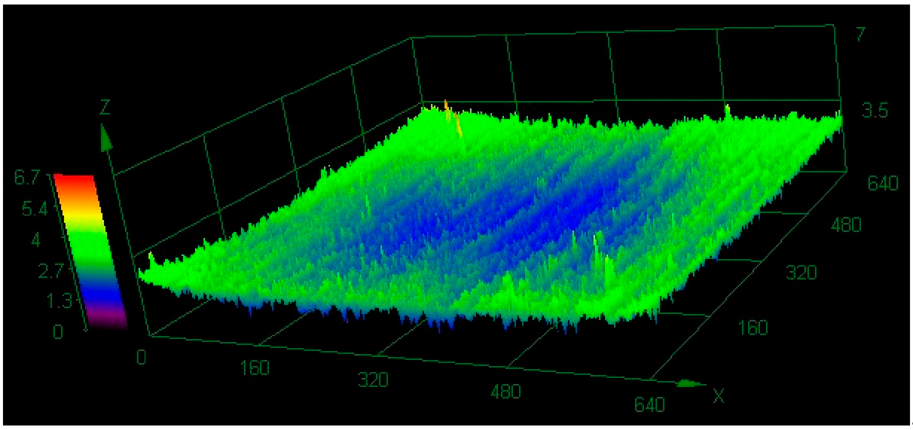

Figure S23. Surface topography of Ti6Al4V sample obtained after treating it with the sandpaper of 2000 gradation (magnification ×426).

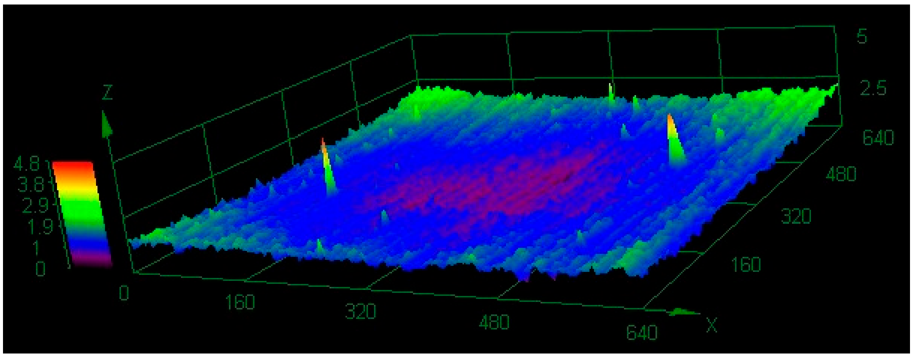

Figure S24. Surface topography of Ti6Al4V sample obtained after treating it with the sandpaper of 1500 gradation (magnification ×426).

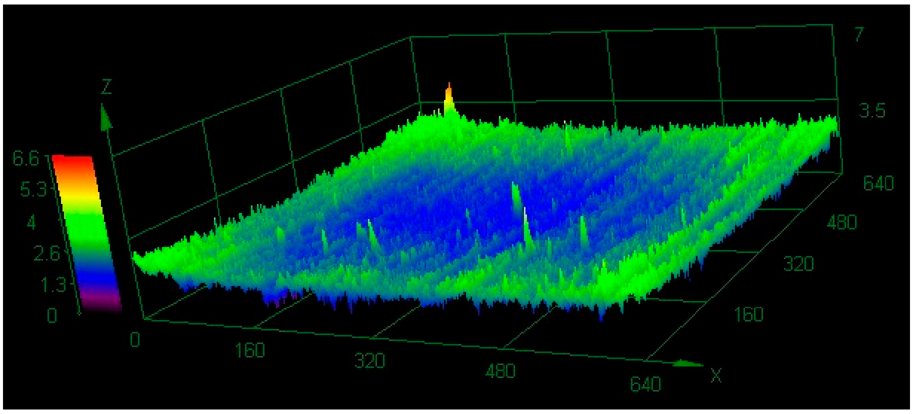

Figure S25. Surface topography of Ti6Al4V sample obtained after treating it with the sandpaper of 1200 gradation (magnification ×426).

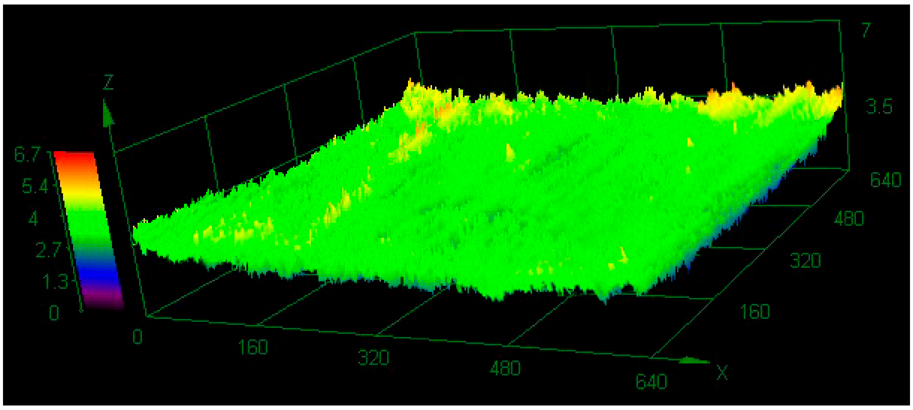

Figure S26. Surface topography of Ti6Al4V sample obtained after treating it with the sandpaper of 1000 gradation (magnification ×426).

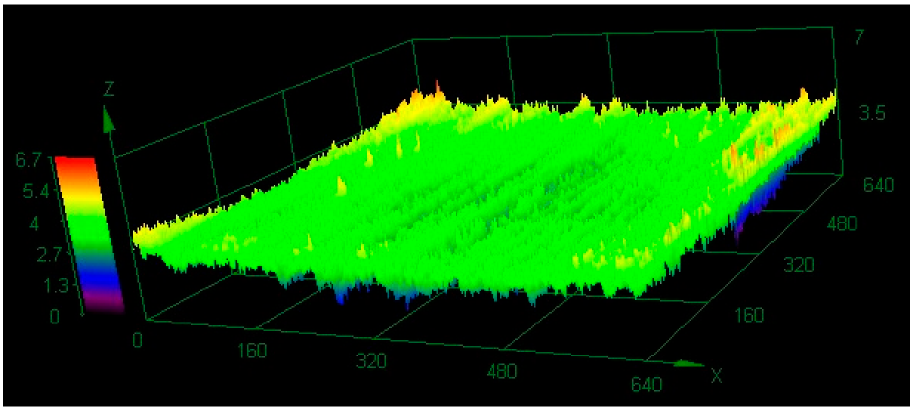

Figure S27. Surface topography of Ti6Al4V sample obtained after treating it with the sandpaper of 800 gradation (magnification ×426).

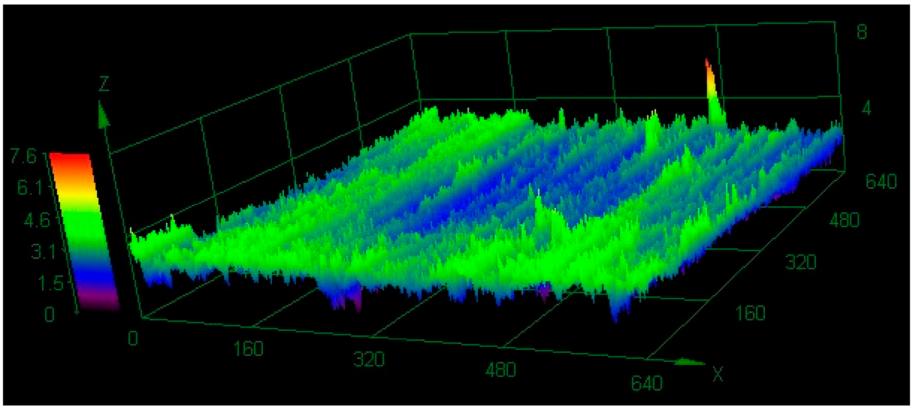

Figure S28. Surface topography of Ti6Al4V sample obtained after treating it with the sandpaper of 600 gradation (magnification ×426).

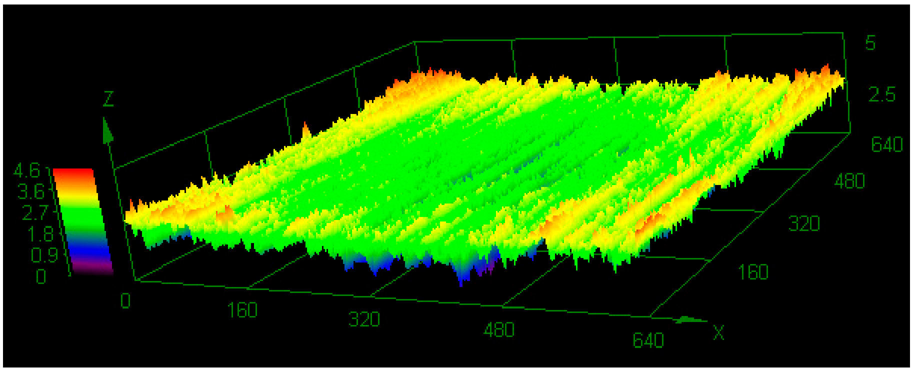

Figure S29. Surface topography of Ti6Al4V sample obtained after treating it with the sandpaper of 400 gradation (magnification ×426).

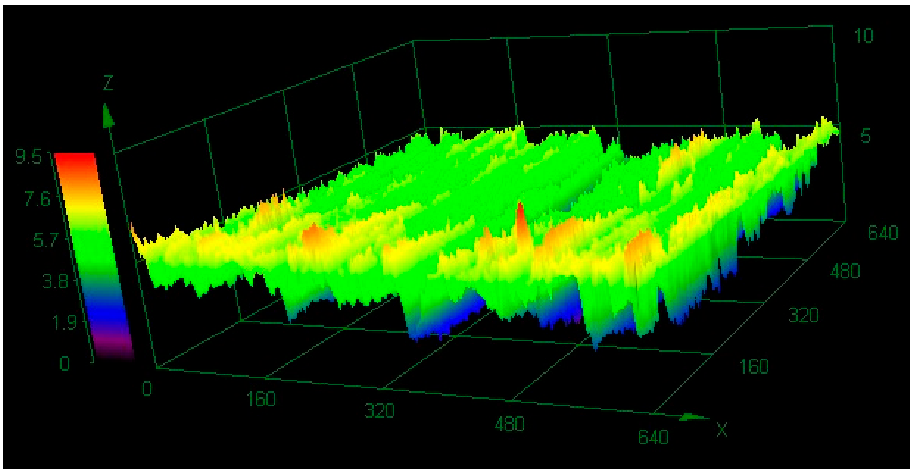

Figure S30. Surface topography of Ti6Al4V sample obtained after treating it with the sandpaper of 240 gradation (magnification ×426).

© 2019 by the authors. Submitted for possible open access publication under the terms and conditions of the Creative Commons Attribution (CC BY) license (http://creativecommons.org/licenses/by/4.0/).
